# Spatial and temporal activity patterns among sympatric tree-roosting bat species in an agriculturally dominated great plains landscape

**DOI:** 10.1371/journal.pone.0286621

**Published:** 2023-06-02

**Authors:** Christopher T. Fill, Craig R. Allen, John F. Benson, Dirac Twidwell

**Affiliations:** 1 Nebraska Cooperative Fish and Wildlife Research Unit, School of Natural Resources, University of Nebraska-Lincoln, Lincoln, Nebraska, United States of America; 2 Center for Resilience in Agricultural Working Landscapes, School of Natural Resources, University of Nebraska-Lincoln, Lincoln, Nebraska, United States of America; 3 School of Natural Resources, University of Nebraska-Lincoln, Lincoln, Nebraska, United States of America; 4 Department of Agronomy and Horticulture, University of Nebraska-Lincoln, Lincoln, Nebraska, United States of America; University of Oklahoma Norman Campus: The University of Oklahoma, UNITED STATES

## Abstract

In agroecosystems, bats can provide a critical ecosystem service by consuming night-flying insect pests. However, many bats also face intense population pressures from human landscape modification, global change and novel diseases. To better understand the behavioral activity of different bat species with respect to space, time, habitat, and other bat species in this environment, we investigated species correlations in space and time over row crop agricultural fields. We used acoustic grids to document spatial and temporal co-occurrence or avoidance between bats and recorded eight species across the 10 field sites we sampled. All species significantly overlapped in two-dimensional space and displayed considerable temporal overlap during the night, yet often exhibited significantly different temporal activity patterns, suggesting fine scale partitioning behavior. Conversion of land to agriculture is likely to increase globally, making it critical to better understand how bat species interact with one another and the landscape to facilitate persistence in these human altered ecosystems.

## Introduction

Interactions between species in space and time are an important driver of both population and community dynamics, especially in human-altered landscapes [1]. The conversion of natural habitats by humans for agricultural production changes patterns of resource availability [2], which may also influence spatiotemporal interactions between closely related species as they compete to fill similar, and likely limited, productive niche space. Some bats provide ecosystem services in agricultural landscapes by consuming insects [3] and exhibit a variety of foraging behaviors and morphological adaptations that could reduce species competition and facilitate coexistence [4–6].

Chiroptera is one of the most species-rich mammalian orders, with members capable of navigation through flight and echolocation [5, 7]. These navigation abilities have been found to influence the behavior and foraging methods of different bat species [4, 8, 9], and can predict not only habitat tendencies, but species vertical niche in airspace as well [10]. Among insectivorous bats, aerial hawking species rely on echolocation to navigate and catch airborne prey while in flight [7, 11]. These species have morphological traits and echolocation call structures for hunting in open, edge, and background-cluttered spaces [4, 5]. Other species use echolocation for patrolling high clutter areas, and are maneuverable enough to glean insects off surfaces as well as in flight [4, 7, 12], however some bats may instead select habitats based on insect distributions [13].

While morphological and acoustic differences can serve to reduce interspecific competition, there is often still potential for species interaction, and certain environments may necessitate partitioning behavior when shared resources are limited. Desert bats have been found to partition time and space when visiting water holes [14, 15] despite an increase in activity as water availability decreased [16]. In other studies, bats in arid environments did not exhibit food partitioning [17], nor spatiotemporal partitioning, despite high overlap and patchy food sources [18]. Even in diverse landscapes bats have been found to partition time [19], especially in areas of greater bat activity [20], and within nights [21]. Differences in diet preferences can also result in morphologically similar bats segregating not only space [13], but also habitat structure [22]. Emergent diseases such as white-nose syndrome, and human activities such as logging and agriculture can also influence bat community structure as well as spatial and temporal partitioning patterns [21, 23, 24].

Here, we examined the spatial and temporal relationships between foraging insectivorous bat species in an agricultural landscape for evidence of partitioning behavior. Activity by bat species in the area has been found to be spatially non-random over row crop fields, with all species favoring areas of tree cover despite available airspace and insect biomass [25]. Intensification of agriculture is an accelerating phenomenon that creates large monocultures that can virtually eliminate native habitats [26–28], limiting options for some wildlife, but allowing for the range expansion of others [29, 30]. We hypothesized spatial and temporal partitioning would occur among bat species across our sampling sites, because forest fragments are a limited resource in extensively farmed landscapes and should increase species interaction potential. Accordingly, we expected a higher degree of activity variation with high clutter species.

## Materials and methods

### Study area

**Fig 1:**
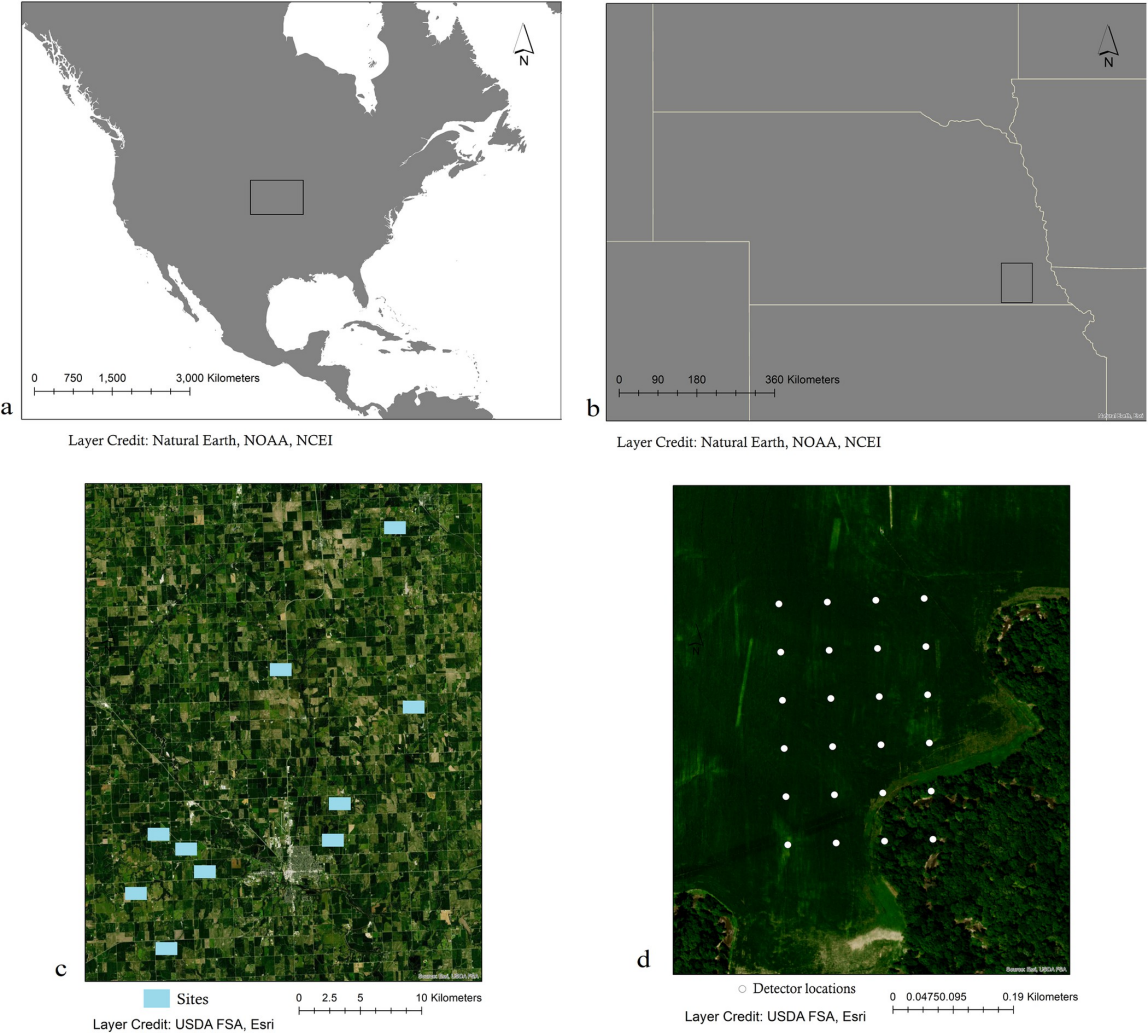
Study area. (A) Study region within the USA, (B) sampling area located in southeast Nebraska, USA, (C) individual field sampling site locations in corn and soybean land uses, (D) detector grid sampling setup within each field site location.

We conducted this research across 10 crop fields in Gage and Lancaster counties in rural southeast Nebraska, USA (Fig 1). These agricultural fields were privately owned, heavily managed by different landowners for the production of corn and soybean, and separated by county roads that crossed in intervals spanning 1.60 km x 1.60 km sections. The area was characterized by large tracts of open farmland on flat upland plains interspersed with riparian buffers, windbreaks, and patches of mature lowland forest. We selected six crop fields that were bordered by some form of tree cover or water source. We also included four fields with little to no nearby forest habitat. With sampling permission granted by each private landowner, no approval permits were required.

### Data collection

We used AnaBat Express passive zero cross acoustic detectors (Titley Scientific, Brendale, QLD, Australia) to record bat activity. We set detectors to a 100 percent recording rate and a trigger setting for echolocation calls above 8 kHz, which units recorded in zero-cross format. We then placed each detector 100 m apart to form a transect grid that bordered the habitat feature and extended into the crop field, spanning a detection area 400 m by 600 m that employed 24 detectors [31]. We entered the coordinates generated in ArcGIS for these locations into hand-held Garmin GPSMAP 64 units for actual detector placement in the field.

We mounted each detector on a modified painter’s pole extended 4 m above the ground, so all units cleared any crop cover for increased recording quality [31] and positioned detectors such that every unit’s omnidirectional microphone faced into the open and away from tree clutter. We left detectors at each deployment field for four consecutive nights, with each detector set to begin recording from at least 30 minutes before sunset until 30 minutes after sunrise. If there was sustained heavy rain, low temperatures, or high winds during a deployment session, we left detectors for additional nights as needed, until four sampling nights were obtained. Issues with battery life occurred at one field, so we redeployed problem detectors and used a combination of different nights, but with as many identical nights as possible across detectors. We sampled from 20 June through 22 August in 2019, when bats are most active in Nebraska, including seven fields in July, two in June, and one in August. Lactating females of most species in the area have been found into July, and some as late as August [29].

### Acoustic analysis

We used AnalookW software (Titley Scientific) to convert raw acoustic detector files to individual bat call files, i.e., a sequence of at least five echolocation pulses, for further analysis. We then analyzed these call files with Kaleidoscope v5.4.9 with the Bats of North America 5.4.0 classifier set to “+1 More Accurate” (Wildlife Acoustics, Concord, MA, USA) to analyze all files and minimize misidentifications. We used Kaleidoscope’s signal parameters as follows: 8–120 kHz frequency range, 1–10 ms length of detected pulses, 500 ms inter-syllable gap, 5 minimum number of pulses, and activated the advanced signal processing feature, which enhances signals for cleaner output. Under the Bats of North America 5.4.0 classifier, we included the following species in the identification process: big brown bat (*Eptesicus fuscus*), eastern red bat (*Lasiurus borealis*), hoary bat (*Lasiurus cinereus*), silver-haired bat (*Lasionycteris noctivagans*), little brown myotis (*Myotis lucifugus*), northern long-eared myotis (*Myotis septentrionalis*), evening bat (*Nycticeius humeralis*), and tricolored bat (*Perimyotis subflavus*). Kaleidoscope Pro looks for unique characteristics in the acoustic calls that makes the identification process of calls to species more feasible, and estimates the presence likelihood of a particular species, ranging from 0 (likely) to 1 (unlikely). We used a cutoff probability value of 0.15 when evaluating species presence at each site, and manually removed all call files incorrectly marked as bat calls. By summing up the numbers of identified acoustic bat call files, and precise recording times, we obtained indices, or relative amounts of species activity, by detector location in time.

### Statistical analyses

We used total number of bat call files pooled from four nights at each field for all analyses. We created a spatial correlation matrix to investigate relationships between bat species occupying shared two-dimensional space across all fields. Since call files of most bat species data were not normally distributed and contained outliers, we used the non-parametric Spearman Rank Correlation method to conduct these species pairwise correlation comparisons with the R package *rcorr* (3.6.2, R Core Development Team). We also constructed a generalized linear mixed model for each response variable (number of call files by a species) using the R package *lme4* to determine the influence of distance from tree cover and the number of call files from the other bat species present, with sampling points within each field as nested random effects (bat 1 ~ distance from trees + bat 2 + bat 3 + bat 4 + bat 5 + 1|field\sampling point). We rescaled the predictor variables of distance from vegetation and species calls in each species-specific model, fitting each model with a negative binomial distribution to account for data overdispersion, and used the R package *MuMIn* to calculate model R^2^ values.

Additionally, we used the R package *overlap* [32] to investigate temporal activity patterns of bats and the degree of any overlap between species. We first estimated and plotted the non-parametric kernel density of detections during nocturnal periods for each bat species, lasting from one half hour before sunset to one half hour after sunrise, over the aggregated 40 total night period. From these plots we then estimated the amount of overlap in temporal activity between pairs of species using a coefficient of overlap ranging from no overlap to complete overlap (0 to 1) and generated 95% confidence intervals through bootstrapping [33]. We used the recommended temporal overlap estimator of Δ4 and smoothing factor of 1 for larger observation counts, and set 10,000 bootstrap samples for each species comparison [32]. Since this coefficient is a descriptive measurement [34], we also calculated Watson’s non-parametric two sample U^2^ statistics to test for significant differences in species temporal activity patterns. This test is often used to determine whether two or more circular distribution datasets are homogenous [35–37].

## Results

During 40 nights of recording, we most frequently detected big brown bats (9,637 call files) and eastern red bats (3,897 call files), along with hoary bats (3,039 call files), evening bats (3,016 call files), and northern long-eared bats (334 call files). Due to the low presence likelihood (p≥0.15) for silver-haired, little brown, and tricolored bats, these species were not included in any subsequent analyses.

### Spatial correlation

**Fig 2:**
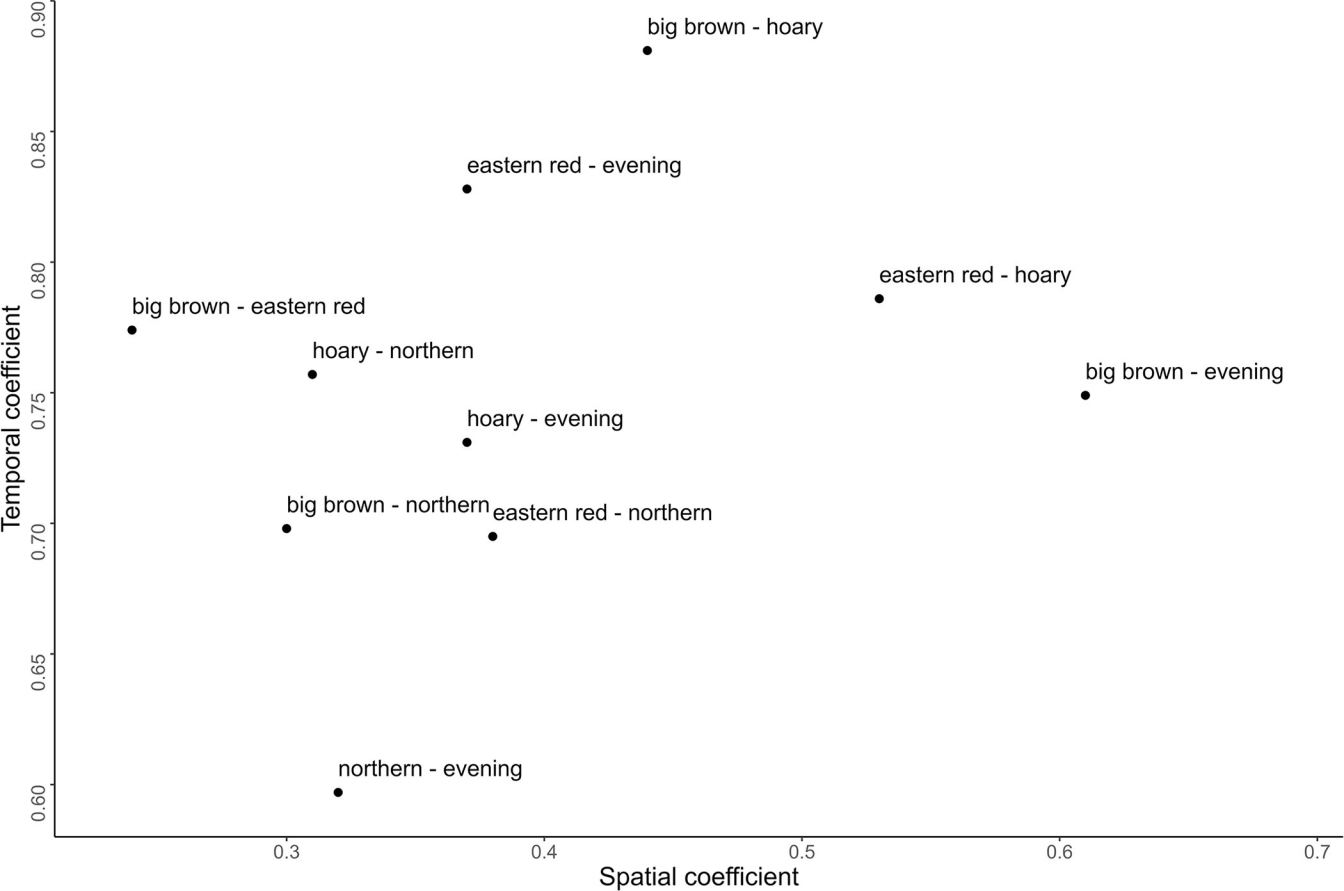
Spatiotemporal overlap between bat species. Spatial pairwise comparisons and temporal overlap coefficients of activity patterns by each bat species across agricultural sampling locations in southeast Nebraska, USA. Points denote amounts of overlap in activity between each species relative to space and time.

Across sites, all bat species exhibited significant overlap in two-dimensional space (r_s_) (Fig 2). Northern long-eared bats, a cluttered space gleaner and hawker, occupied some of the least amount of this space with all other bat species (r_s_ = 0.30–0.38), as did big brown bats with eastern red bats (r_s_ = 0.24), and evening bats with both hoary and eastern red bats (r_s_ = 0.37). All other bat species were more positively correlated in space, with the most overlap occurring between big brown and evening bats (r_s_ = 0.61), and eastern red bats with hoary bats (r_s_ = 0.53). Generalized linear mixed models suggested that spatially, the activity of each species was significantly negatively affected by distance to tree cover, with northern long-eared and eastern red bats the most adversely influenced, followed by evening and big brown bats (Table 1). In terms of pairwise species interactions, only eastern red bats were significantly negatively correlated in space with northern long-eared bats; the activity of most other species was positively associated spatially (Table 1). Hoary and big brown bats were the most positively correlated in two-dimensional space, followed by evening and northern long-eared bats (Table 1).

**Table 1:** Spatial associations in activity of different bat species and forest cover.

β	Big brown (R^2^ = 0.59)^b^	Eastern red (R^2^ = 0.40)	Hoary (R^2^ = 0.04)	Northern (R^2^ = 0.55)	Evening (R^2^ = 0.05)
Forest (distance)	-0.453 ***	-0.964 ***	-0.321 *	-1.251 *	-0.465 **
Big brown	-----	0.427 **	0.334 **	0.388	0.486 **
Eastern red	0.482 ***	-----	0.245.	0.585	0.601 ***
Hoary	1.264 ***	0.631 **	-----	0.341	-0.070
Northern	-0.009	-0.502 ***	0.077	-----	0.069
Evening	0.636 ***	0.619 ***	-0.252	0.999 ***	-----

Beta coefficients (β) for fixed predictor variables and model fit (R^2^) of bat species activity patterns from generalized linear mixed models fitted with negative binomial distributions. Individual species models were constructed using a single species as the response variable, and distance from forest cover and species call counts as rescaled predictor variables, with sampling points within sites as nested random effects. Bat calls were recorded over 10 corn and soybean fields in southeast Nebraska, USA.

^a^Significance codes:

*** [0, 0.001]

** (0.001, 0.01]

* (0.01, 0.05],. (0.05, 0.1], ‘‘ (0.1, 1]

^b^R^2^: marginal R^2^ species model fits

### Temporal correlation and overlap

**Fig 3:**
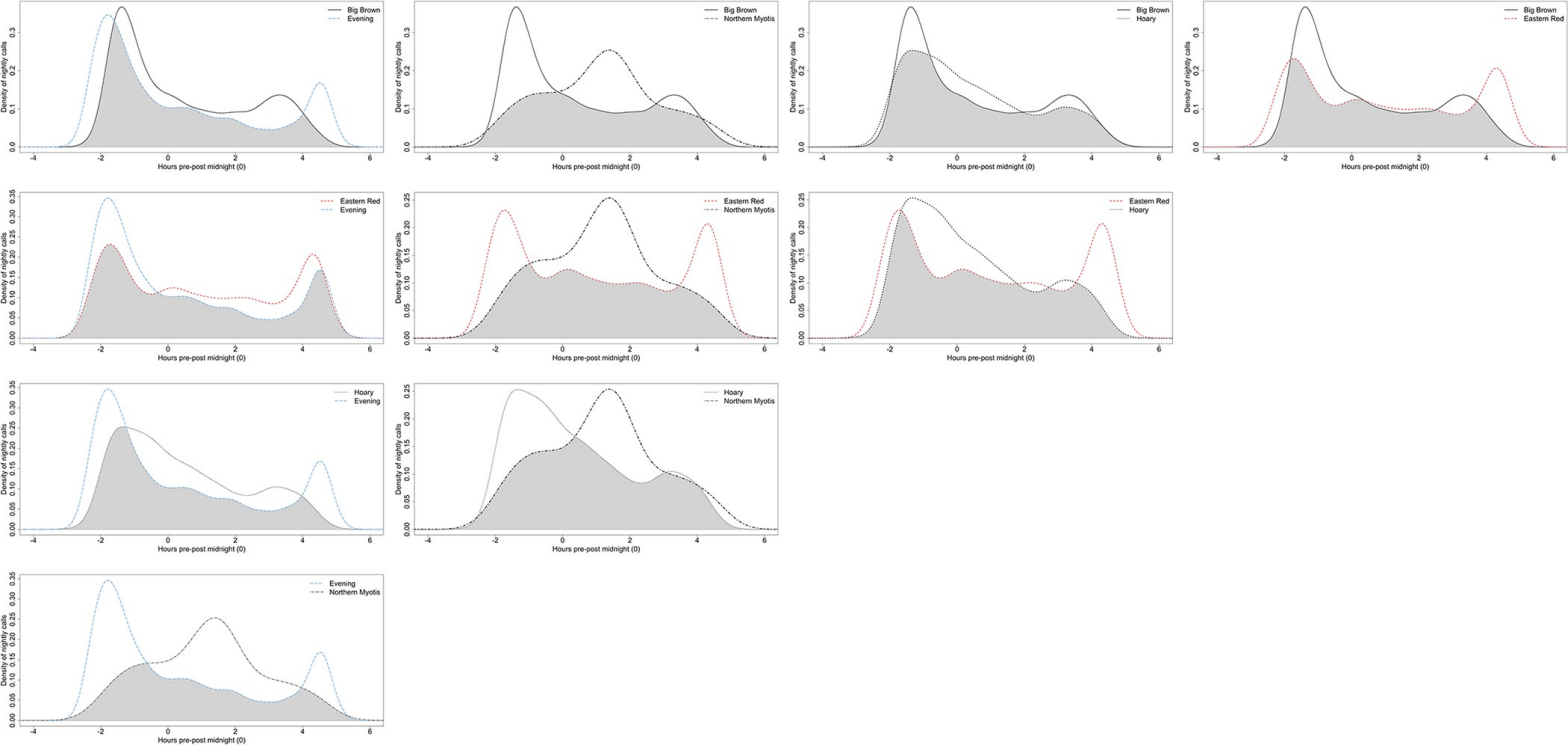
Kernel density estimations of bat temporal activity patterns. Density estimates of the nocturnal activity patterns by each bat species with the other bat species present across agricultural sampling locations in southeast Nebraska, USA. The shaded area in each plot represents the temporal overlap coefficient, with the x-axis centered around midnight.

Most nightly bat activity patterns displayed a bimodal distribution with the first and highest peak shortly after sunset, and a second lesser peak before sunrise (Fig 3). Density plots also indicated considerable temporal overlap between species, with all pairwise overlap coefficients between Δ = 0.59–0.88 (Table 2). Northern long-eared bats had the lowest temporal overlap (Δ < 0.70) with all other aerial hawking species except hoary bats (Δ = 0.76, CI: 95% 0.71–0.80), while having comparatively moderate to lower levels of spatial overlap (r_s_ < 0.4) with all species (Fig 2). Eastern red bats exhibited the most amount of temporal overlap with most other species, including evening bats (Δ = 0.83, CI: 95% 0.81–0.85), hoary bats (Δ = 0.79, CI: 95% 0.76–0.81), and big brown bats (Δ = 0.77, CI: 95% 0.71–0.75); only big brown bat temporal overlap with hoary bats (Δ = 0.88, CI: 95% 0.86–0.89) was higher. Hoary bat was the only species detected before sunset and appeared to increase temporal overlap with all species as distance from forested areas increased (Table 2). Big brown, eastern red, and evening bats were first detected at or after sunset, while northern long-eared and all other bat species were not recorded until after sunset. Northern long-eared bats temporally overlapped the most with all species near forested areas, while big brown bats maintained relatively consistent temporal overlap with evening and eastern red bats, both of which decreased temporal overlap with increasing distance from forested areas (Table 2). Watson’s U^2^ tests indicated all pairs of species exhibited significantly different activity patterns, suggesting temporal partitioning behavior (all p≤0.05; Table 2).

**Table 2:** Temporal correlations between species activity patterns.

Bat species	Overlap (Δ)	95% CI	Δ Near	Δ Mid	Δ Far	U^2^ Test Stat^a^
big brown–hoary	0.881	0.86–0.89	0.827	0.903	0.899	3.27
eastern red–evening	0.828	0.81–0.85	0.809	0.831	0.755	6.22
eastern red–hoary	0.786	0.76–0.81	0.779	0.771	0.813	12.68
big brown–eastern red	0.774	0.71–0.75	0.751	0.729	0.748	17.33
hoary–northern	0.757	0.71–0.80	0.758	-----	-----	2.21
big brown–evening	0.749	0.73–0.76	0.736	0.721	0.741	15.61
hoary–evening	0.731	0.70–0.75	0.703	0.728	0.813	12.94
big brown–northern	0.698	0.65–0.74	0.669	-----	-----	3.43
eastern red–northern	0.695	0.65–0.74	0.737	-----	-----	3.76
northern–evening	0.597	0.55–0.64	0.596	-----	-----	5.89

Watson U^2^ statistical tests and temporal coefficients of overlapping (Δ) between bat species activity patterns across all sampling points, as well as overlap values for points near (≤100 m), mid (100–300 m), and far (≥300 m) from forest habitat. All data was collected in row crop fields in southeast Nebraska, USA to evaluate resource partitioning; all U^2^ values were significant (p≤0.05).

^a^Critical value = 0.187

## Discussion

Our data suggest that in the agriculturally dominated landscape of southeast Nebraska, bat species exhibited fine-scale partitioning behavior while occupying similar airspace along crop fields. While we found substantial amounts of spatial and temporal overlap among bat species in general, only northern long-eared bats and eastern red bats were significantly negatively correlated in two-dimensional space, and all species exhibited significantly different temporal activity patterns from one another. Given the habitat, energetic, and temporal constraints of these nocturnal and small-bodied mammals, it is likely our data are indicative of fine-scale spatiotemporal niche partitioning behavior in this increasingly common row crop landscape setting.

Bat activity of all species in this study declined with distance from tree cover (Table 1), a behavioral trend consistent with previous research with bats in this area and landscape type [25, 38]. Northern long-eared bat activity was affected the most of all species in this regard, which was expected, since this species primarily forages in high-clutter environments and is reliant on forested habitats [4, 39, 40]. While other bat species seemed less affected, they have also been found to roost in trees and other woody and man-made structures [41–43], and will forage in closed forests, forest edges, and open areas [4, 5, 8], likely lessening the negative effects of limited forest availability in this landscape setting. The apparent negative spatial correlation between eastern red bats and northern long-eared bats may represent competition for forest edge spaces, since both species were much less active in open areas (Table 1). It may also have been at least partially the result of mistaken identification between the similar acoustic calls by both species. Some studies in more forested landscapes have found that post white-nose syndrome, when northern long-eared bat populations have declined, eastern red bat activity slightly decreased or remained stable rather than increasing [24, 44], which may indicate these two species do not regularly compete for resource niche space when suitable habitat is available.

We found little support for spatial partitioning between bat species, as all appeared to overlap considerably in two-dimensional space (Fig 2). While this is likely an effect of habitat availability, with bats prioritizing activity along forested areas, the variation in spatial overlap may represent finer scales of partitioning in two-dimensional space, especially when combined with temporal activity patterns (Fig 2). Alternatively, the apparent lack of spatial partitioning may be due to a more opportunistic feeding approach in which bats optimize their foraging strategy [17] or are adapted to patchy food sources [18]. Daily weather conditions tend to affect insect distribution in agricultural landscapes [45], with wind often blowing smaller insects to sheltered areas, like windbreaks and other forest types [46], likely contributing to more bat foraging activity in those habitats.

Our temporal overlap analysis suggested that all bat species in our study exhibited significantly different temporal activity patterns from one another (Table 2), supporting the hypothesis that temporal partitioning would occur among the bat species in our study area. This is likely an effect of the considerable amounts of two-dimensional space shared between the species in our study, allowing for co-use of limited foraging habitat. However, despite these differences, all species still shared moderate to high temporal overlap throughout the night (Table 2), suggesting that temporal partitioning occurs at a finer scale. In terms of activity patterns, the single Myotis species we recorded exhibited a markedly different activity pattern than the other species, peaking almost two hours after midnight when the activity of all other species declined (Fig 3), and supporting our hypothesis that high-clutter species would experience a higher degree of activity variation. Other studies in agricultural environments have found this species to peak around sunrise and sunset [21], however these nightly patterns can be highly variable [47], and may be a result of landscape features, weather conditions, prey availability, or temporal partitioning. Since smaller high-clutter species like Myotis, which typically eat smaller insects [8] and may have fewer alternative prey items to consume, are likely more easily excluded by the larger bat species, they may be forced to adjust their activity levels to when the other bats traverse and forage wider areas. Big brown, eastern red, and evening bats exhibited considerable overlap in their temporal activity patterns, and all possess robust jaws for consuming hard-shelled insects as well as moths [48, 49], suggesting these species may exploit similar insect prey when temporally available. While we were unable to quantify food availability, other studies have found that similar bat species partition time, lessening the need to partition space when feeding [16, 19].

The relatively high temporal overlap between species (Δ = 0.59–0.88) may seem contradictory to the statistical differences in pairwise temporal activity patterns we documented. An alternative explanation to the interpretation that bat species in our study area exhibit fine-scale temporal partitioning is that these statistical differences may not reflect biological significance. We used all acoustic data for these tests, since acoustics do not account for individual bats, but rather lend insight into relative amounts of species activity. Thus, we acknowledge that it was difficult to control for the lack of independence between bat detections within species, some of which likely came from the same individuals. The lack of independence may have artificially inflated the statistical power of these pairwise tests; however, differences in temporal activity between species can also be visualized through the kernel density plots (Fig 3). It is also possible that the relatively high temporal overlap measurements should be evaluated in a different light than studies of cathemeral species that are more active throughout a 24-hour period, for instance coyotes and other large mammalian predators [50], since potential interactions between nocturnal bat species are more constrained temporally. We also recognize that automated identification programs designed to classify bat species from echolocation calls have low agreement between them [51] and the wide variation in call structures makes many species difficult to distinguish [52].

While the bats in our study seemed to be partitioning space at finer scales, they also rely on echolocation to navigate, and those with similar call bandwidths may additionally partition acoustic space and shift call frequency to avoid acoustic jamming [53, 54]. The social information that bats can glean from the echolocation calls of other individuals may also explain, in part, differences in species activity patterns [55]. Bats are known to utilize upper airspace out of range of ground-based detectors [56–58], and likely partition this vertical space as well [10]. Additionally, vegetation structure can also influence how different bat species occupy shared niche spaces in forested habitats [59–61].

Our data provide some insight into how multiple tree roosting bat species co-utilize agricultural fields through apparent fine scale spatiotemporal partitioning behaviors. Since most of the species in our study area are negatively affected by loss of tree cover and rely on woody structures for daytime roosts, especially threatened Myotis species [40], we recommend the preservation of forest fragments and riparian areas as foraging habitat for these species. As fragmented environments increase globally, more research is needed to better understand how bat species interact with one another, the landscape [62, 63], and their insect prey [64, 65], allowing for more effective conservation so that bats and other wildlife might persist in and continue to benefit these human-altered ecosystems.
